# Pediatric-Adapted Liking Survey (PALS): A Diet and Activity Screener in Pediatric Care

**DOI:** 10.3390/nu11071641

**Published:** 2019-07-18

**Authors:** Kayla Vosburgh, Sharon R. Smith, Samantha Oldman, Tania Huedo-Medina, Valerie B. Duffy

**Affiliations:** 1 Department of Allied Health Sciences, University of Connecticut, Storrs, CT 06269, USA; 2 CT Children’s Medical Center, University of Connecticut School of Medicine, Hartford, CT 06106 2, USA

**Keywords:** dietary screener, obesity prevention, sweet preference, children, diet quality

## Abstract

Clinical settings need rapid yet useful methods to screen for diet and activity behaviors for brief interventions and to guide obesity prevention efforts. In an urban pediatric emergency department, these behaviors were screened in children and parents with the 33-item Pediatric-Adapted Liking Survey (PALS) to assess the reliability and validity of a Healthy Behavior Index (HBI) generated from the PALS responses. The PALS was completed by 925 children (average age = 11 ± 4 years, 55% publicly insured, 37% overweight/obese by Body Mass Index Percentile, BMI-P) and 925 parents. Child–parent dyads differed most in liking of vegetables, sweets, sweet drinks, and screen time. Across the sample, child and parent HBIs were variable, normally distributed with adequate internal reliability and construct validity, revealing two dimensions (less healthy—sweet drinks, sweets, sedentary behaviors; healthy—vegetables, fruits, proteins). The HBI showed criterion validity, detecting healthier indexes in parents vs. children, females vs. males, privately- vs. publicly-health insured, and residence in higher- vs. lower-income communities. Parent’s HBI explained some variability in child BMI percentile. Greater liking of sweets/carbohydrates partially mediated the association between low family income and higher BMI percentile. These findings support the utility of PALS as a dietary behavior and activity screener for children and their parents in a clinical setting.

## 1. Introduction

The worldwide childhood overweight/obesity prevalence ranges from 22 to 24% [1]. Obesity in U.S. children is estimated at 17%, including 5.8% extreme obesity (BMI ≥ 120% of the 95th percentile) [2]. Obesity prevention requires a multi-sector approach [3], including screening, brief interventions and referrals between clinical and community sectors [4]. As the pediatric emergency department (PED) is utilized for non-urgent care [5], it should be part of this multi-sector approach [6,7,8,9] to reach low-income children who often have unhealthy dietary behaviors and lack access to primary care [6]. Brief obesity interventions have been successfully accomplished in the PED [7]. Clinicians need rapid, yet useful tools to screen behaviors for patient-centered interventions to promote healthy behaviors [10]. As parent involvement is critical [11], these tools should capture parent and child behaviors.

Conventional dietary assessment asks children or parents to recall food/beverage intake (e.g., 24-h recall,) or usual intake frequency [12,13], which is time intensive, often involves misreporting [14], and may cause defensive parent response and low-compliance in a clinical setting [15]. Screening usual consumption by asking likes/dislikes offers a feasible alternative. Recall of liking is quicker and cognitively simpler than behavioral recall with potentially less parent unease. Reported food liking correlates with reported intake [16,17,18], biomarkers of intake and/or adiposity in children [18] and adults [19,20,21]. The Pediatric-adapted Liking Survey (PALS) is fast, has a high response rate in the PED, with good-to-excellent clinical-to-home test–retest reliability [22]. Furthermore, results from an assessment of children’s preference for food and physical activity (PA) can guide program planning [23]. Health promotion across the socio-ecological framework needs to develop healthy food and PA preferences in children [24].

The present study further develops PALS [22] to address needs in clinical settings. One need is to screen dietary behaviors in children and their parents (i.e., child–parent dyads) with comparable methods. The dietary patterns of children and parents can show weak-to-moderate resemblance [25]. The second is to assess dietary behaviors toward food/beverage groups and diet healthiness (i.e., diet quality). Few studies have examined diet quality in child–parent dyads [26]. We have shown that liking survey responses can form a reliable and valid diet quality index that explains significant variation in markers of nutritional status and health in preschoolers [18] and adults [21,27]. Diet quality indexes improve the understanding of diet-health relationships [28], inform interventions [29] and monitoring [30] in children. From analysis of three cycles of U.S. National Health and Nutrition Examination Survey (NHANES), diet quality among children is low, showing socio-economical and race/ethnic disparities [31]. The third need is to feasibly screen PA and sedentary behaviors in child–parent dyads. PA encouragement is key as children age, especially targeting those of economic disadvantage [32]. As questionnaires inform PA assessment [33], we enhanced the PALS [22] with physical and sedentary activities as well as additional foods.

Our specific objective was to screen both children’s and parent’s food and activity liking and to assess the reliability and validity of a Healthy Behavior Index (HBI) generated from the liking responses. Measures of reliability and validity followed that for the Healthy Eating Index [34], including the ability of the HBI to detect differences between child and parent, by the child’s age and gender, proxies of the family’s economic status, and the child’s Body Mass Index Percentile (BMI-P). Finally, we examined models of interaction between income and food liking to explain variability in the child’s BMI-P.

## 2. Materials and Methods

### 2.1. Participants

This observational study enrolled a convenience sample of 5 to 17-year-old children who sought medical care at the Connecticut Children’s Medical Center’s PED in Hartford, CT. The sample size was to capture diversity in the child to address the study aims and allow for multivariate analysis within a diverse sample. Children were excluded from participating if they had a history of severe behavioral/mental health conditions, were non-English speaking, or too ill to participate. Institutional Review Boards approved this study. To participate, parents/guardians signed informed consent, and children ages 7 and older signed an assent. Of those consenting to participate and meeting the inclusion criteria, 93% completed the protocol. The final sample, collected from March 2013 to April 2016, included 925 child–parent dyads who were diverse in child age, race/ethnicity, and family economic status (Table 1).

### 2.2. Study Procedure and Measures

Data collection took place in the patient’s exam room. Research assistants enrolled patients, confirmed the inclusion/exclusion criteria, collected the child’s address, age, gender, race/ethnicity, type of health insurance, and history of chronic medical condition (e.g., asthma, diabetes).

The community of family residence by zip code was reported by the parent/caregiver which served beyond type of health insurance as another proxy of family income and level of food insecurity. Median household income by zip code, reported by the U.S. Census Bureau, 2010–2014 American Community Survey 5-Year Estimates, was used to determine the family’s income level. A Connecticut ranking of town food security (based on economic and social characteristics, access to food retailers, utilization of public food assistance) was used to assess participants’ risk of food insecurity [35].

Pediatric-Adapted Liking Survey (PALS): Both child and parent/guardian were asked to complete the PALS, a food and activity liking/disliking survey, based on their own likes and dislikes (average completion time was <4 min). This three-page, paper/pencil PALS consisted of 33 food items and activities, represented with both pictures and words as described previously [22]. Participants reported their level of liking/disliking, marking a perpendicular line anywhere along the scale with seven faces labeled as “love it,” “really like it,” “like it,” “it’s ok,” “dislike it,” “really dislike it,” and “hate it.” Distance was measured from the scale center (0; “he/she thinks it’s okay”) to the participant’s marking (±100; “he/she loves/hates it”). Children and parents/caregivers also could mark “never tried/done.”

The Healthy Behavior Index (HBI) was conceptually constructed based on the 2015 Dietary Guidelines [36], with a single index similar to the Healthy Eating Index (HEI) and following our previously validated, liking-based diet quality indices [18,21] with the addition of PA and screen time (sedentary behavior). Foods and activities were sorted into conceptual groups and multiplied by weights consistent with the Dietary Guidelines [18,21,27]: vegetables (+3), fruits (+2), protein (+2), sweets (−3), sugary drinks (−3), fiber (+2), salty (−2), dairy (+2), PA (+2) and screen time (−3). The final HBI was the average of weighted groups that formed an internally reliable, normally distributed index: vegetables, fruits, protein, sweets, sugary drinks, and screen time. Higher indexes indicated healthier behaviors.

Measured and Self-Reported Adiposity: The child’s height was measured by trained research assistants (cm; portable Stadiometer, Seca^®^) and weight was obtained from the electronic health record (kg; platform medical scale) to calculate Body Mass Index (BMI). Age-and-sex specific BMI-Ps were calculated with the with the online calculator [37], with the child’s exact age (based on birth and measurement dates) and the U.S. Centers for Disease Control 2000 growth charts to assign underweight <5th, healthy weight 5th–<85th, overweight 85th–<95th, or obese ≥95th percentile [38]. Parents/caregivers and children self-reported the child’s body size using a sex-specific, 7-point drawing [39] for categorization (underweight <2, healthy weight 2 to <5, overweight 5 to 6, obese ≥ 6).

### 2.3. Data Analysis

Data were analyzed using SPSS statistical software (version 22.0) with the Process v3.1 (afhayes@processmacro.org) with a significance criterion of *p* < 0.05. Descriptive statistics were used to compare BMI-P against national statistics and contrast measured versus self-rated body size. All variables were evaluated for distribution, normality and central tendency. Table 2 describes the assessment of reliability and validity of the HBI. Analysis of covariance included controlling for demographic variables (age, gender, race/ethnicity, income, as appropriate) as indicated in the results section. Direct relationships between parent and child HBI and adiposity were examined with standard multiple linear regression analysis while controlling for demographic variables and child’s liking of PA. Additionally, multivariate modeling was used to assess associations between food liking, proxies of family income and food insecurity, and child BMI-P.

## 3. Results

Overall, 37.4% of children were classified as overweight or obese by BMI-P (Table 3), which was comparable to the U.S. average of 36.6% of children aged 5 to <18 years old [2]. Children ages 9 to 13 years old had higher rates of overweight (21%) and obesity (25.3%) than any other age group. Extreme obesity in children ages 6–11 and 12–19 years old was 7 and 9.5%, respectively, and exceeded U.S. averages of 4.3 and 9.1%, respectively [2]. Independent of age and gender, a higher BMI-P was seen in children covered by public health insurance (70.02 ± 1.26 SEM) than by private health insurance (62.64 ± 1.52) (F(1,916) = 14.231, *p* < 0.001). In similar analyses, higher BMI-P was seen in children from families who reported residency in communities with lower income (compressing the highest and low income levels (F(2,894) = 5.583, *p* < 0.005) and greater risk of food insecurity (F(3,901) = 3.574, *p* = 0.014). Among overweight children, nearly half of children (47.6%) and parents (49.7%) self-reported being a lower body size than measured; among obese children, most children (94.8%) and parents (84.4%) also self-reported being a lower body size than measured.

### 3.1. Relative Comparison of Parent and Child Food and Activity Liking

**Figure 1:**
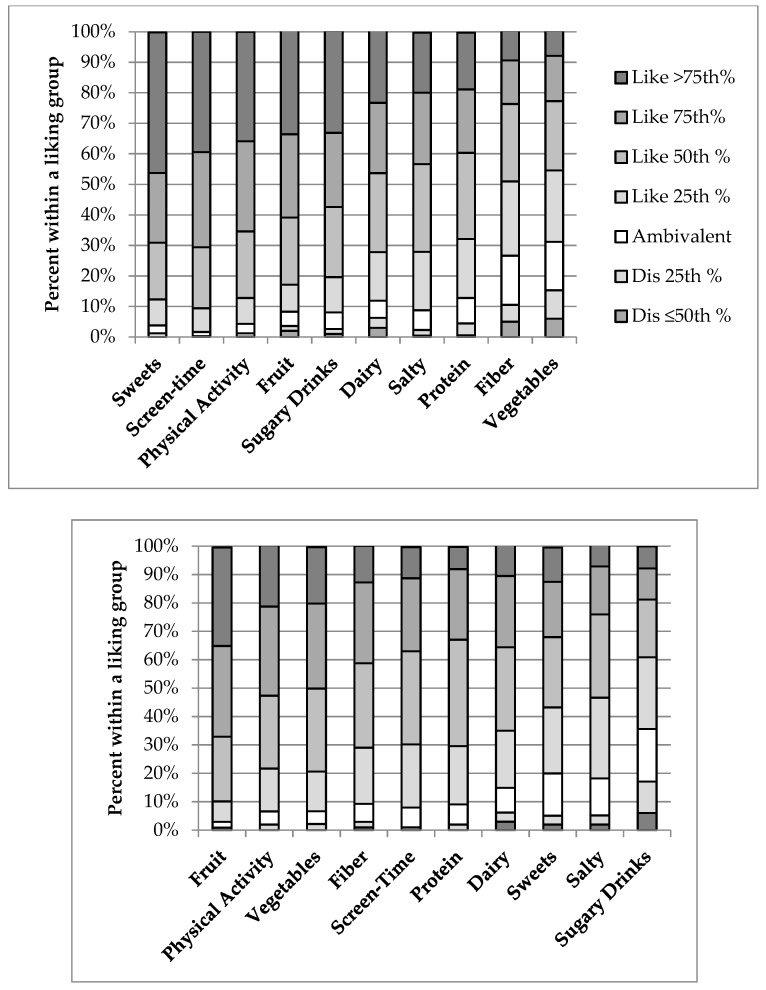
Liking of food/beverage and activity groups (left to right as most to least liked) in children (top graph) and parents (bottom graph), shown as percent within a food or activity group as liking (above the white neutral rating) and disliking (below the white neutral rating), with the darker the shading indicating stronger the liking or disliking.

Across the sample (Figure 1), parents averaged the highest preference for fruits and PA, while children reported the highest preference for sweets and screen time (e.g., watching TV, playing video games, listening to music). Children reported lower liking for fiber-rich foods and vegetables compared with parent reporting. Variance within food/activity groups was highest for children’s liking of healthier groups (vegetables, fruit, proteins), and parental liking of the less healthy groups (sweets drinks and sweets) (Table 4). For children and parents, the least liked items had the highest variability in ratings. By effect sizes, the magnitude of difference between child–parent dyads was largest for vegetables, sweet drinks, screen time, and sweets.

Following our previous study [18], three groups of children were identified from the relative liking for sweets versus a pleasurable non-food: greater liking of screen time than sweets; equal liking; greater liking of sweets than screen time. From ANCOVA controlling for age and gender, children with higher affinity for screen time than sweets had significantly higher BMI-P [F(2, 873) = 4.022, *p* < 0.05] than children with higher affinity for sweets than screen time.

### 3.2. Internal Reliability of the HBI

The parent and child HBI approached acceptable internal reliability (α = 0.646 versus 0.613, respectively). Children and parents who reported high liking of sweets also reported significantly higher liking of screen time and sugary drinks, as well as lower liking (disliking) for vegetables (all Spearman’s rho’s, *p* < 0.01). Child and parent HBI were highly influenced by liking of vegetables, sugary drinks, and sweets (Pearson’s r between ±0.47 and 0.71, *p* < 0.01).

### 3.3. Construct Validity of the HBI

**Figure 2:**
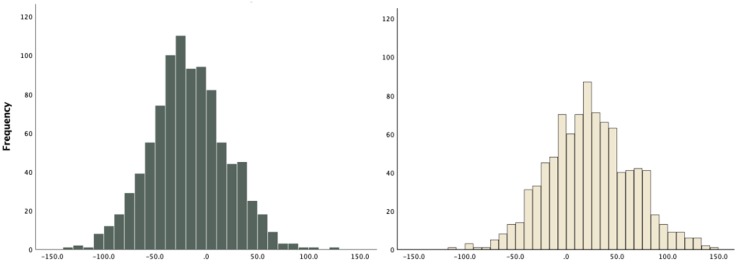
Histograms showing normal distributions of HBI in children (5–17 years old; left) and parents (right).

The child and parent HBI were normally distributed (Figure 2), with the parent’s distribution towards the higher indexes. Although weak, child and parent HBI were significantly correlated (r = 0.219, *p* < 0.01), with similar correlation across all groups making up the HBI.

Principal component analysis (PCA) of the child HBI revealed two underlying dimensions—less healthy (screen time, sugary drinks, sweets) and healthy (vegetables, fruits, protein), which accounted for 57.2% of total variance. The PCA for the parents as well as child demographic and BMI-P categories (shown in Table 2) produced similar results for less healthy and healthy dimensions and >50% total variance explained, supporting a consistent underlying structure of the HBI.

### 3.4. Concurrent Criterion Validity of the HBI

The comparison of mean differences in child HBI via ANOVA, with post-hoc tests as appropriate, revealed significant effects of gender (males < females), health insurance type (public < private), race/ethnicity (Hispanic/Latino and Black/African American < White), income levels (determined through zip code analysis; low income < high income), and risk of food insecurity (high risk < low risk) (Table 5). Similar findings were seen for child or parent HBI. Greater age was correlated with healthier behaviors (r = 0.239, *p* = 0.000) as seen in females and males. In an income by race/ethnicity ANCOVA controlling for age and gender, income category was the sole significant contributor to child HBI (*p* < 0.001), with only a trend for an interaction with race/ethnicity (*p* = 0.09). In a gender by race ANCOVA, controlling for age, there were significant main effects on child HBI (*p* = 0.008 and 0.014, respectively), but no significant interaction effects. In summary, children who were older, white, female, covered by private insurance, and from communities with higher income and lower risk for food insecurity had the highest or healthiest HBI.

No significant differences in child HBI were found with BMI-P categories. However, a multiple linear regression model predicting child BMI-P from parent HBI, gender, insurance, and child liking for PA was significant among children of healthy weight (between 10th and 85th BMI-P). Significant predictors of higher child BMI-P were seen among lower parent HBI (β = −0.11, *p* < 0.05) and higher child liking of PA (β = 0.15, *p* < 0.005).

**Figure 3:**
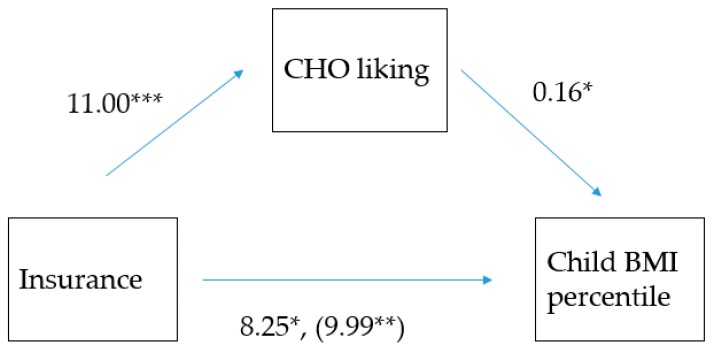
Model of the association between health insurance as a proxy of family income (dichotomous variable, where 1 = private; 2 = public) and the child’s Body Mass Index Percentile (BMI-P), mediated by the parent’s liking of carbohydrate-rich foods among 925 in children seen in an urban pediatric emergency department. * *p* ≤ 0.05; ** *p* ≤ 0.01; *** *p* ≤ 0.005. The (coefficient in the parenthesis) represents the association between insurance and BMI-P before parent liking was added to the model, indicating that parent liking partially mediated the insurance and BMI-P relationship (indirect coefficient = 1.954, *p* = 0.05).

Due to the interactions between health insurance (proxy of family income), parent liking and BMI-P, the possibility that parent-liking mediates the relationship between health insurance and child BMI-P was examined. Of several models tested, parent liking for carbohydrate-rich foods (average of salty, sweet drinks, fiber, sweets groups; Cronbach’s α = 0.74), was most explanatory, particularly in younger children (5 to 9 years old). Shown in Figure 3, higher parent liking of these foods explained some of the correlation between public insurance and higher child BMI-P (Z = 1.954, *p* = 0.05; bootstrap lower level confidence interval = 0.2239, bootstrap upper level confidence interval = 3.6938).

## 4. Discussion

Clinical settings need brief measures to screen children’s behaviors in a method that is acceptable to families, has reasonable utility, can guide child and family-centered messages to encourage healthy behaviors, and can inform interventions for the prevention of obesity, particularly in at-risk groups. The present observational study recruited children and families from an urban, pediatric emergency department (PED) to assess children’s and parents’ liking of foods and activities with the Pediatric-Adapted Liking Survey (PALS) and test the reliability and validity of a Healthy Behavior Index (HBI), constructed from their PALS responses. The study sample of 925 child–parent dyads was diverse in race/ethnicity and >50% low-income, with children ranging in age from 5 to 17 years old, and with rates of overweight and obesity at or exceeding that for the U.S. The PALS is a novel diet and activity screener that showed good acceptability in this clinical setting and diverse sample. It was easily completed by children and parents and identified expected differences (children reporting a greater affinity for sugary foods/beverages and screen time, but lower affinity for vegetables than parents). The HBI neared adequate internal reliability and had normal distribution across both parents and children. For validity, the HBI measured two themes (healthy and less healthy), supporting its construct validity, and detected expected differences in healthy behaviors between groups, supporting its criterion validity. Healthier indexes were seen in females versus males, older versus younger children, parent versus child, families on private versus public insurance, and those living in higher income/food secure versus lower income/food insecure communities. For Body Mass Index Percentile (BMI-P), a higher parent-reported HBI was associated with lower percentiles across children who fell in the healthy range (between the 10th and 85th percentiles). In sub-analysis, part of the association between higher child BMI-P among families on public health insurance (i.e., lower income) was explained by greater parent liking of carbohydrate/sweet foods and beverages.

Simple indices with low participant and practitioner burden, such as the PALS and generated HBI, can be useful in a clinical setting [40] and to assess changes in response to interventions for children and families [30]. The indexes emphasize that positive health arises from moderating less healthy behaviors and encouraging those that are healthier. As the most effective obesity prevention programs for children involve the family [11], clinicians could begin conversations based on similarities and differences between child and parent dietary/activity likes and dislikes [41]. Parents influence the child’s consumption of healthy and less healthy foods through controlling their availability, modeling consumption of these foods, and setting norms and attitudes toward healthy eating [42]. In the present study, child–parent dyads differed most for liking of sweets and sugary beverages. This agrees with a large multi-center study of families, which found stronger associations between parents and children for healthy rather than unhealthy foods [43]. Children prefer higher level of sweets than adults, linked to physical growth and energy need during development [44]. As higher added sugar consumption associates with poor diet quality and excess adiposity [45], families can look to healthier sweet options including fruit and fruit-based desserts. Parenting behaviors of restricting less healthy foods, using foods as a punishment or reward, or pressuring children to eat are ineffective at improving healthy eating behaviors [42]. Child–parent dyads also differed significantly in vegetable liking. Clinicians can encourage parents to show explicit liking of healthier foods [42], including tasting and consuming a variety of vegetables, involving children in cooking, supporting school meal participation, and family mealtime. Parents and children differed significantly in liking screen time yet were closer in liking for PA. Parent’s liking and knowledge about screen time is significantly related to levels of screen time activity in children, which supports screen time interventions that target the child and the parent [46]. Parent modeling and support, including co-activity, can improve PA in children [47]. As preferences, attitudes and believes of parents are important predictors of PA that is performed with parents and children together [48], clinicians could probe beyond the PALS screening to identify which activities are enjoyed by both the child and parent and ways to facilitate and encourage family-based PA.

The indexes derived from the PALS showed good variability across the sample with acceptable internal reliability and validity. Although Cronbach’s alpha for the HBI fell below the traditionally accepted value of α = 0.70, this may be expected due to the complex nature of measuring diet quality, and therefore may not be a required characteristic [34]. Additionally, the child and parent HBI had a similar multi-dimensional structure of healthy (fruits, vegetables, protein) and less healthy (sweets, sweet drinks, and screen time) items. The HBI showed concurrent criterion validity through distinguishing between groups with known differences. Our results and others have found higher diet quality and health behavior indexes among females [49]. However, our findings that older children had higher diet quality and health behavior indexes differed from others, which found the opposite age relationship [50,51]. The present study found that white children reported higher diet quality and health behaviors than Hispanics/Latinos, consistent with an analysis of 2003–2004 U.S. NHANES [50], yet no significant difference was found between Blacks/African Americans and Hispanics/Latinos [50]. Finally, by using proxies of family income from community demographics, lower HBIs were found among children from families with lower income, receiving public medical insurance, and living in communities at high risk for food insecurity, consistent with multiple studies [50,51,52]. Our results are comparable to previous work that find healthier diet quality and behaviors among parents than their children [53].

The value of diet quality and health indexes is the ability to associate with health outcomes [40]. The present study found a significant but weak association between healthier parent HBI and lower BMI-P in healthy weight children. However, the child-reported HBI did not associate significantly with BMI-P. The association between indexes of diet quality or health behaviors and adiposity in recent scientific literature has been inconsistent. In cross-sectional studies, it has ranged from better diet quality and higher adiposities among children [54], to no significant association [55,56,57] or lack of consistent association [58], to healthier diet patterns in those who were overweight or obese [59]. Other studies only report demographic differences in diet quality in children and not the diet quality–adiposity association [60,61]. However, a large prospective study in children found significant associations with less healthy diet quality and increased adiposity over time [62]. Regarding activity, obese children have higher reported screen time and lower PA than do non-obese children from a systematic review, but the differences are small [63]. In the present study, the lack of significant association between the child-reported HBI and BMI-P may reflect a higher level of misreporting among overweight/obese children. Weight status has been shown to influence dietary reports by children, with heavier children being more likely to misreport due to social pressures and expectations [64]. It also may be important to examine dietary components to improve the diet quality and lower energy intakes, such as sugary beverages [65], fruits and vegetables [57] or, as in the present study, carbohydrate/sweet foods. Additionally, improvements in diet quality have been associated with improvements in body composition across an intensive diet and lifestyle intervention for overweight/obese adolescents [66]. According to a critical review, improvements in diet quality are required to improve cardiometabolic health, including obesity [67].

We found a positive association between child-reporting liking of PA and BMI-P, which is consistent with finding that obese children were more likely to report taking part in healthy behaviors [68]. Obese children are more likely to have been informed of their weight status by a physician [68], despite their perception of lower body size than what is measured in this study and others [69]. In the present study, children reported a high liking for screen time. Higher liking of screen time activities in children has been shown to associated with greater screen time behaviors [46]. Excessive screen time has been linked to lower diet quality [70], increased rates of obesity and negative health conditions [71]. When compared to liking for sweets, we found that children with a relatively higher affinity for screen time than sweets had significantly higher BMI-P than those who preferred sweets to screen time. Examining these relative rankings could help tailor messages to support healthy behavior and healthy weight. Parental encouragement has shown positive longitudinal effects on PA in adolescents [72].

Despite the findings of the present study, the question remains whether it is useful to ask parents and children to self-report their diet and PA behaviors. Furthermore, can asking likes or dislikes of foods/beverages and activities be reflective enough of usual behaviors to serve as a screener to guide a dialogue between health professionals, children and families? Screeners are short instruments to, for example, distinguish between healthier versus less healthy behaviors. Behavioral screeners need to be useful but not overly burdensome or cause families to become defensive [15]. All self-reported measures have the potential for reporting bias yet supply important information despite the emergence of dietary intake biomarkers [73]. If over time, for example, we eat what we like and avoid what we do not, reported liking reflects a pattern of what was consumed, but cannot capture total energy intake. Taste and food preference drive consumption. Food preference and intake are used interchangeably in nutrition literature [74,75] and food preference provides a proxy of consumption for examining health outcomes [76]. Survey-reported preference or liking correlates with self-reported intake in children [18,77] and adults [16,19,27,78,79,80], as well as with biomarkers of dietary intake and/or adiposity in children [18] and adults [19,20,21]. Similar to the present study, liking survey responses can be formed into an index of diet quality that explains variability in carotenoid status in preschoolers [18] and cardiovascular disease risk factors and BMI [21,27] in adults. However, food preference or liking can show marginal [19] or non-significant [81,82] associations with self-reported intake or BMI. Discrepancy in reported liking and intake does not imply that reported liking is inaccurate, and instead may reflect dietary restraint (intake is less than liking) in adults [19,27,83] and parents who are trying to limit their children’s consumption of less healthy foods [18]. Conversely, individuals who are trying to improve their diet healthiness may consume a food that is not well liked [18,27].

Encounters between health professionals and families can motivate attention to and action towards improving a child’s healthy behaviors for obesity prevention [84]. Improvements in the healthiness of a child’s diet is promoted when both parents and children prefer the same food/beverage [85]. The PALS in the present study has previously identified patterns of food preferences that are associated with parent feeding practices [86]. Having children and parents self-evaluate their food and activity liking can act as a stepping stone to introduce a conversation regarding healthy behaviors and to identify goals and areas for change. Even if children are not overweight or obese at the time of the assessment, their behaviors may put them at future health risk. It is important to address and improve pleasure from healthy eating to achieve healthier dietary behaviors [17,87,88] and tailor nutrition education messages [89]. Preliminary work from our group has shown that doing the PALS online is acceptable in children and parents, is reported to stimulate self-reflection on diet and activity behaviors, can generate immediate tailored feedback on diet quality and healthy behaviors [90]. Preferences can change, including in response to marketing of unhealthy foods [91] as well as with interventions to improve preference for healthy foods [92,93,94,95] and decrease preference for less healthy foods [83,96].

This study had both strengths and weaknesses. The PED can be an acceptable setting to screen health behaviors related to obesity risk and for brief interventions, particularly because it provides health care to high-risk populations, such as low-income, minority families [6,7,8,9]. Additionally, this study utilized the PALS diet and activity screener, which was acceptable to both children and parents, with testing of reliability and validity using multiple statistical techniques and criteria [34,97]. The PALS was similar in structure and methods to our previous study in preschoolers, which was parent-reported, validated against reported dietary intake, a biomarker of carotenoid status and BMI-P [18,98]. As a limitation, only one measure of dietary behaviors was assessed without a more complete evaluation of PA behaviors. Since obesogenic dietary behaviors involve both hedonic responses to pleasurable foods and appetite, the PALS could be supplemented with constructs of appetite and satiety [99]. Previous work by our group has shown increased precision in making diet-health associations by combining the liking survey with multiple measures of dietary behaviors [18]. Because the intent was to screen for dietary and PA, the present study did not include a biomarker of nutritional status or a device to measure PA. Furthermore, the HBI did not explain BMI-P across children with lowest to highest percentiles, but only among children of healthy BMI-P. Detecting associations between child adiposity, dietary patterns and behaviors may require longitudinal study designs [100]. Further, BMI-P may not be the most useful measure of adiposity for a racially/ethnically diverse sample of children and adolescents [101].

## 5. Conclusions

Pediatric clinical and translational research settings need rapid yet useful ways to screen for health behaviors to inform brief interventions, referrals, and obesity prevention programs. A simple liking survey provides an acceptable and useful screener of diet and activity behaviors in child–parent dyads. The survey took less than 4 min to complete on average and had a high participation rate. Liking for foods and activities was formed into a healthy behavior index that had acceptable internal reliability and good variability across children and parents. Healthier behavior indexes were seen in children from income-disadvantaged families and those from less food secure communities. Liking for less healthy foods explained some of the association between low family income and higher child BMI percentile. Health care providers could use the liking survey responses to initiate conversations with children and parents and to encourage healthy diet and physical activity behaviors. The PALS can be performed as a paper/pencil survey, but for future direction, also can be performed online with theory-based health promotion messages delivered to the children and parents based on response algorithms [90,102]. PALS responses across groups of child–parent dyads can inform broader nutrition education programming and messages, such as in the nutrition education arm of the U.S. Supplemental Nutrition Assistance Program (SNAP-Ed) [103].

## Figures and Tables

**Table 1:** Characteristics of children seeking medical care in a Pediatric Emergency Department.

	*n* = 925	%
**Age (Avg. 10.9 years)**		
5–<9 year	356	38
9–<13 year	257	28
13–17 year	312	34
**Sex**		
Male	463	50.1
Female	462	49.9
**Race/Ethnicity**		
Caucasian	357	38.6
Black	133	14.4
Hispanic	344	37.2
Other	91	9.8
**Insurance**		
Private	382	41.3
Public	507	54.8
Self pay	16	1.7
Other	20	2.2
Income Level *^,a^		
<$21,432	26	2.8
$21,433–41,186	288	31.1
$41,187–68,212	245	26.5
$68,213–112,262	313	33.8
>$112,263	29	3.1
Food Insecurity *^,b^		
Greatest risk	574	62.1
Higher than average risk	102	11
Lower than average risk	134	14.5
Lowest risk	99	10.7

* Percentages ≠ 100 due to missing data (<3%); ^a^ Based on zip code analysis using U.S. Census Bureau data from the 2010–2014 American Community Survey 5-Year Estimates (U.S. Census Bureau. 2010–2014 American Community Survey 5-year estimates: Income in the past 12 months (in 2014 inflation-adjusted dollars)). American FactFinder: Community Facts Website. factfinder.census.gov/faces/nav/jsf/pages/index.xhtml. Accessed May 30, 2019.); ^b^ Based on data from the Zwick Center for Food and Resource Policy and the Cooperative Extension System at the University of Connecticut [35].

**Table 2:** Tests to assess the internal reliability and validity of the Healthy Behavior Index (HBI) [34].

Question	Test Statistic
**Reliability**	
How internally consistent is the total index?	Cronbach’s Alpha
What are the relationships among the index components?	Pearson’s *r* correlations between each component
Which components have the most influence on the total index?	Pearson’s *r* correlations between each component and the total index
**Construct and Concurrent Criterion Validity**	
Does the index score foods and behaviors based on those recommended by the 2015 Dietary Guidelines?	Descriptive statistics
Does the index allow for sufficient variation in scores among individual?	Measures of central tendency, histogram, normality testing (Kolmogorov-Smirnov)
What is the underlying structure of the index (i.e., > 1 dimension)?	Principal component analysis and plot; derived factors to explain >50% of variance
Does the index distinguish between groups with known differences (i.e., concurrent criterion validity)?	Descriptive statistics, ANOVA with post-hoc analysis, ANCOVA, multiple regression analysis between demographic characteristics, PA liking and child’s BMI-P

**Table 3:** Body Mass Index (BMI) percentiles by age and gender of children who were patients at a pediatric emergency department (PED).

	5–<18 Years		5–<9 Years		9–<13 Years		13–<18 Years	
	Count	% *	Count	% *	Count	% *	Count	% *
**5th–<85th percentile**								
Male	275	29.7	102	28.7	74	28.8	99	31.7
Female	277	29.9	110	30.9	59	23.0	108	34.6
Total	552	59.6	212	59.6	133	51.8	207	66.3
**85th–<95th percentile**								
Male	68	7.4	22	6.2	31	12.1	15	4.8
Female	82	8.9	27	7.6	23	8.9	32	10.3
Total	150	16.2	49	13.8	54	21.0	47	15.1
**≥95th percentile**								
Male	105	11.4	48	13.5	35	13.6	22	7.1
Female	91	9.8	28	7.9	30	11.7	33	10.6
Total	196	21.2	76	21.4	65	25.3	55	17.7

* Percentages ≠ 100 due to missing data (Percent of total sample size, *n* = 925; <2% missing). Underweight (<5th percentile) not shown due to small sample size (*n* = 19, avg. age = 9.7 years, mean BMI percentile = 1.52 and SD = 1.33).

**Table 4:** Variance and estimated effect sizes of child (*n* = 925) and parent (*n* = 925) survey-reported liking of foods and activities.

	Child			Parent			Effect Size
	Mean	SD	Variance	Mean	SD	Variance	Cohen’s d
Vegetables	19.5	40.5	1636.6	48.4	30.6	938.3	0.8 *
Fruits	56.9	33.1	1098.0	60.5	27.4	749.7	0.1
Protein	40.9	35.3	1242.7	37.9	27.9	778.6	0.1
Sweet drinks	55.0	33.3	1108.7	14.1	39.6	1565.5	1.1 *
Screen time	64.3	26.5	701.6	39.9	27.7	768.0	0.9 *
Sweets	64.4	31.2	974.2	31.0	36.3	1317.7	1.0 *
Fiber	23.6	38.4	1476.7	41.6	30.6	936.4	0.5
Salty	44.1	32.1	1028.4	28.3	30.6	933.4	0.5
PA	59.5	29.8	888.1	49.3	30.7	940.4	0.3
Dairy	45.6	36.7	1346.3	35.5	34.6	1198.1	0.3

* Large effect size.

**Table 5:** Analysis of variance for mean child and parent Healthy Behavior Index (HBI) by child’s demographics, community food environment, and adiposity.

	Child				Parent			
Characteristic *	Mean HBI	*n*	SD	*p*-Value	Mean HBI	*n*	SD	*p*-Value
**Gender**								
Male	−53.8	449	40.1	0.002 **	13.0	449	44.8	0.280
Female	−45.3	439	43.0		16.2	439	43.9	
**Race/Ethnicity**								
White	−41.1	341	42.3	0.000 **	23.0	341	43.1	<0.001 **
Af. Amer./Black	−55.2	129	39.3	0.006 †	10.1	129	43.3	0.023 †
Hispanic/Latino	−55.5	330	40.7	0.000 †	8.7	330	44.2	<0.001 †
**Insurance Type**								
Private	−44.0	364	40.4	0.001 **	23.7	364	41.3	<0.001 **
Public	−53.7	490	41.9		7.3	490	45.0	
**Income Level**								
$21,433–41,186	−58.9	277	40.9	0.000 **	4.8	277	45.2	<0.001 *
$41,187–68,212	−47.4	234	41.5	0.015 ^a^	14.7	234	41.3	0.075
$68,213–112,262	−41.8	301	41.0	0.000 ^a^	24.4	301	42.3	<0.001 ^a^
**Food Insecurity**								
Greatest risk	−54.2	552	40.9	0.000 **	7.8	552	43.5	<0.001 **
>than avg. risk	−46.1	99	42.1	0.272	19.7	99	46.4	0.058
<than avg. risk	−40.7	125	39.0	0.005 ^b^	27.9	125	39.9	<0.001 ^b^
Lowest risk	−36.8	97	44.6	0.001 ^b^	27.3	97	42.3	<0.001 ^b^
**BMI Percentile**								
Normal weight	−49.6	523	40.7		14.8	523	44.3	
Overweight	−46.6	149	42.4	0.716 ^	12.0	149	40.7	0.767 ^
Obese	−49.0	189	42.7	0.984 ^	15.1	189	44.5	0.996 ^
**Overall**	−49.4	908	42.1	---	14.5	904	43.9	---

* Characteristics of child, not parent; the overall number is less than 925 due to missing data; ** Overall significant result, *p* < 0.05; † Significant result, *p* < 0.05, compared to white; a Significant result, *p* < 0.05, compared to lower income level ($21,43 3–41,186); b Significant result, *p* < 0.05, compared to those at greatest risk for food insecurity; ^ *p*-value compared to normal weight.
